# Epidemiological Features of Human Brucellosis in Iran (2011-2018) and Prediction of Brucellosis with Data-Mining Models

**Published:** 2019-12-04

**Authors:** Hadi Bagheri, Leili Tapak, Manoochehr Karami, Behzad Amiri, Zahra Cherghi

**Affiliations:** ^1^Department of Epidemiology, School of Public Health, Hamadan University of Medical Sciences, Hamadan, Iran; ^2^Department of Biostatistics, School of Public Health, Hamadan University of Medical Sciences, Hamadan, Iran; ^3^Social Determinants of Health Research Center, Hamadan University of Medical Sciences, Hamadan, Iran; ^4^Centers for Communicable Disease Control and Prevention, Ministry of Health and Medical Education, Tehran, Iran; ^5^Modeling of Noncommunicable Diseases Research Center, Hamadan University of Medical Sciences, Hamadan, Iran

**Keywords:** Brucellosis, Iran, Data mining, Cohort studies

## Abstract

**Background:** Brucellosis is known as the major zoonotic disease. We aimed to compare the performance of some data-mining models in predicting the monthly brucellosis cases in Iran.

**Study design:** Population-based cohort study.

**Methods:** Three data mining techniques including the Support Vector Machine (SVM), Multivariate Adaptive Regression Splines (MARS), and Random Forest (RF) besides to one classic model including Auto-Regressive Integrated Moving Average (ARIMA) was used to predict the monthly incidence of brucellosis in Iran during 2011-2018. We used several criteria (root mean square error (RMSE), mean absolute error (MAE), coefficient of determination (R2 ) and intra-class correlation coefficient (ICC) for appraising the accuracy of prediction and performance of our models. All analysis was done using free statistical software of R3.4.0

**Results:** Overall 118867 cases (with a mean age of 34.01±1.65 yr) of brucellosis were observed and seven-year incidence rate of brucellosis in Iran was 21.78 (95% CI: 21.66, 21.91). The majority of patients (58.84%) were male and 25-29 yr old. The first three provinces with the highest incidence rate of brucellosis included the following; Kurdistan (71.39 per 100,000), Lorestan (68.09 per 100,000) and Hamadan (56.24 per 100,000).

**Conclusion:** Brucellosis was more common in males, 25-29 aged yr, western provinces and spring months. The disease had a decreasing trend in the last years. MARS model was more appropriate rather than data mining models for prediction of monthly incidence rate of brucellosis.

## Introduction

Brucellosis is known as the major zoonotic disease. Therefore, the prevalence of brucellosis in humans is highly correlated to the prevalence among animals. It is widely distributed in both humans and animals, around the world, especially in developing countries^1^.

According to WHO around 500,000 people are infected by the brucellosis annually and the portion of eastern Mediterranean (EMRO) was around 45,000 cases per year. On the other hand, due of the variety of symptoms of the disease in humans and the problems that occur in the diagnosis of the disease, its prevalence in the world is less than the actual outbreak and the incidence and prevalence rate of brucellosis are underestimated so that, only one-fifth of cases is detected^2^. Although brucellosis has declined in many countries in the world because of the implementation of control and eradication programs, human brucellosis remained as a public health concern in Iran yet. According to the recent national study (during 2013-2015) the prevalence of brucellosis was 15.4% in Iran^3^.

Despite the high incidence of disease in Iran, the time trend of it has been declining^4^. Moreover, brucellosis in Iran is a native disease and the share of rural areas is more than an urban area. Brucellosis has been most prevalent in the western and northwestern parts of Iran and has borne a considerable economic burden^5^. Due to the long-term complications of brucellosis and, the economic losses, control and timely precautions of brucellosis are very important ^1, 6, 7^.

There are several statistical models in the field of medical science that can predict the incidence of diseases ^8, 9^. The use of these models in health care data has led to an appropriate model for predicting and using those data in the epidemiological health care system and the rapid and accurate determination of epidemiologists' timely and appropriate healthcare decisions.

Recently, a machine-based time-series model has been developed to effectively predict the incidence and predictive problems of infectious disease modeling frequencies. These techniques are powerful tools because they do not need to estimate the classical class presumptions and consider the nonlinear effects of relationships as well as the interactions between them^10^.

In the present study, three data- mining techniques of the Support Vector Machine (SVM), Multivariate Adaptive Regression Splines (MARS) and Random Forest (RF) time series and one classic approach as ARIMA (Auto-Regressive Integrated Moving Average.) models were utilized to predict the monthly incidence of brucellosis in Iran.

The purpose of this study was to compare the performance of the four SVM, RF, MARS and ARIMA methods in predicting the monthly number of human brucellosis cases in Iran during 2011-2018, in order to identify a model that results in better prediction used by general health in control and prevention of future pandemics.

## Methods

### Study area and data source

This national time-series study was conducted in Iran. Iran is a country in the southwest of Asia with over 81 million inhabitants, and 18th most populous country. Iran is subdivided into 31 provinces. The monthly data of brucellosis cases, including *Abortus* and *Melitensis* species of *Brucella* registered in the Ministry of Health and Medical Education of Iran (MOHME) was used from 2011 (Mar/Apr) to 2018 (Feb/Mar).

### Implementation of models

Three data- mining techniques of the Support Vector Machine (SVM), Multivariate Adaptive Regression Splines (MARS) and Random Forest (RF) time series and one classic approach as ARIMA (Auto-Regressive Integrated Moving Average.) models were utilized to predict the monthly incidence of brucellosis in Iran.

A brief description of the models was presented. To implement the three data-mining models, the data was split into two subsets. For this purpose, 80% of the data (Mar 2011 to Feb/Mar 2014) was used as the training set, and the remaining 20% (Jan 2015 to Feb/Mar 2018) was considered as the test set.

Then each model was fitted and trained by the data in the training set. Then predictions were obtained from the data in the test sets. The performance of the models was assessed by comparing the predicted and observed values of the monthly incidence of brucellosis using several criteria (root mean square error (RMSE), mean absolute error (MAE), coefficient of determination (R^2^) and intra-class correlation coefficient (ICC)), these criteria was explained in more details in fourth further section.

### Support vector machine (SVM)

Support vector machine as a one of the technique of data mining, is supervised machine learning algorithm used in classification and regression problems^9^

In this study, radial based function (RBF) was recruited. The SVM has three tuning parameters maximized. Trial and error method was used to tune the parameters of the SVM using 10-fold cross-validation on the training data^9^.

### Random Forest (RF)

An RF or random decision forests as a nonparametric statistical method (free model) is a classification and regression tool^11-13^. In regression problem and with the aim of improving the predictive performance RF combines the predictions obtained from a large incidence of regression trees where each tree is created using recursive partitioning input space until similar or homogenous subspaces^11-14^.

The monthly incidence rate (per 100,000) of brucellosis was considered as the response variable and monthly incidence rate incidence of brucellosis in the previous 12 months (X_1_,…,X_12_) as well as the season, year and month were considered as predictors.

To implement the random forest algorithm and achieve the best model, we used trial and error. Threfore, we tried 3 to 8 mtry and 100 to 1000 trees.

In RF model, a bootstrap random sample and some of predictors (mtry) were used for creating each trees. Two parameters (ntree and mtry), had been tuned in order to avoid overfitting problem. We achieved the best RF model, with 3 to 8 mtry and 100 to 1000 trees.

To achieve optimization of the model, cross-validation method was used, so that for training test data by 5-fold cross-validation where the training data were randomly divided into 5 sets. Then a single subsample of the k subsamples (20%) is considered as the validation data for testing the model and to refine model, and the remaining k-1 (80%) data was used for training data. This process is then iterated k times and the k results are then averaged.

For identify which covariates have the most impact on outcome variable, a statistic of variable importance criterion (VIC) was calculated according to the contribution of predicators in constructing a tree. Here, VIC was calculated using the percent increase in MSE, which is the mean decrease of accuracy in predications for out of bag samples by removing a given variable from the model^9^.

### Multivariate Adaptive Regression Splines (MARS)

MARS as one of the data mining technique is a form of regression analysis as well as it is a modification of classification and regression tree method^14^. MARS model can be applied in prediction of continuous outcomes without considering any assumptions about the underlying functional relationship between explanatory variables (previous 12-month observations, year, season and month) and outcome of interest (in our study; monthly incidence rate of brucellosis)^15^. In present time-series study, the general form of MARS model can be considered as follows:

$$\begin{array}{l} y_{t}=\beta_{0}+\beta_{1}\max(0,X_{1}-c_{1})+\cdots +\beta_{12}\max(0,X_{12}-c_{12})\\ +\beta_{13}\max(0,Year_{}-c_{13})+\beta_{14}\max(0,Season-c_{14})\\ +\beta_{15}\max(0,month-c_{15}) \end{array}$$

In the above equation *β_i_* shows regression coefficients related to the covariates and *c_i_* shows knots (some constant). This model allows to include all possible interactions between the covariates (e.g. *β_ij_* max(0,X_i_-c_i_)max(0,X_j_-c_j_ indicates the interaction between X_i_ and X_j_) ^15, 16^.

### Evaluating criteria

We used several criteria for appraising the accuracy of prediction and performance of our models (SVM, RFTS and MARS), which included the root mean square error (RMSE), mean absolute error (MAE), coefficient of determination (R^2^) and intra-class correlation coefficient (ICC).

The RMSE and MAE are the metric of goodness-of-fit according to high incidences and moderate incidences, respectively. The smaller values of both mentioned metrics indicate a better prediction performance. R^2^ or the coefficient of determination measures the amount of linear relationship between observed and expected incidences and contains the values between 0 and 1. ICC evaluates the agreement percent between predicted and observed incidences and encompass the range of -1 to 1. The greater values of R^2^ and ICC show a better consistency between the observed and predicted values. The formulas of the three criteria are as follows as:

$$\begin{array}{l} RMSE=\sqrt{\frac{1}{n}\sum_{i=1}^{n}{\left(y_{observed}-y_{predicted}\right)}^{2}}\\ MAE=\frac{1}{n}\sum_{i=1}^{n}\left|y_{observed}-y_{predicted}\right|\\ R^{2}=\frac{\sum_{i=1}^{n}{\left(y_{predicted}-y_{mean}\right)}^{2}}{\sum_{i=1}^{n}{\left(y_{observed}-y_{mean}\right)}^{2}} \end{array}$$

Where n is the number of observed value of incidence rate, y is the incidence values and y_mean_ is the average of incidence rate values.

### Software

All analysis was done using free statistical software of R version 3.2.5 (2016-04-14) with the packages including e1071 version 1.7.1 (2016-11-26), Random Forest version 4.6.14 (2018-03-22) and earth version 5.1.2 (6.9.2019).

## Results

In this national study (2011-2018), overall 118867 cases (with a mean age of 34.01±1.65) of brucellosis were observed and a seven-year incidence rate of brucellosis in Iran was 21.78 per 100,000 (95% CI: 21.66, 21.91). The majority of patients (58.84%) were male. Eighty-nine percent were 25-29 yr old. Brucellosis from infected sheep (compared to other livestock) to humans was the most common (63%) type of transmission mode (Table 1).

**Table 1:** The characteristics of monthly incidence of Brucellosis cases in Iran from 2011-2018

**Year**	**Cases**	**Ir** ^a^ **± SD**	**Mean of age (yr)**	**Gender** **(% of males)**	**Age group with highest (yr)**	**Percent of livestock**				
						**Sheep**	**Goat**	**Cow**	**Buffalo**	**Camel**
2011	11,056	14.7±3.96	35.11 ±0.37	56.09	15-24	63.27	25.12	11.07	0.29	0.25
2012	15,700	17.2±5.23	34.09 ±0.48	59.45	25-29	63.61	24.99	10.88	0.28	0.24
2013	18,527	20.0±6.92	32.64± 0.58	61.45	25-29	62.68	26.11	10.71	0.27	0.22
2014	20,206	22.0±7.17	34.32 ±0.89	58.76	25-29	62.80	26.28	10.45	0.26	0.21
2015	20,192	21.4±7.57	32.55 ±1.01	59.61	25-29	41.28	14.03	42.69	1.19	0.80
2016	15,274	15.9±4.25	32.65 ±0.24	58.36	25-29	41.96	14.31	41.78	1.17	0.79
2017	16,027	16.5±4.52	36.37 ±1.68	58.43	25-29	42.61	14.53	40.90	1.17	0.79
2018^b^	1,885	16.2±1.61	37.09 ±0.00	57.76	>65	52.07	19.72	26.95	0.75	0.52
**Total**	118,867	27.2±4.12	34.01 ±1.65	58.84	25-29	63.27	25.12	11.07	0.29	0.25

^a^ Incidence Rate (IR) per 100,000 ^b^ January to Februar

**Figure 1:**
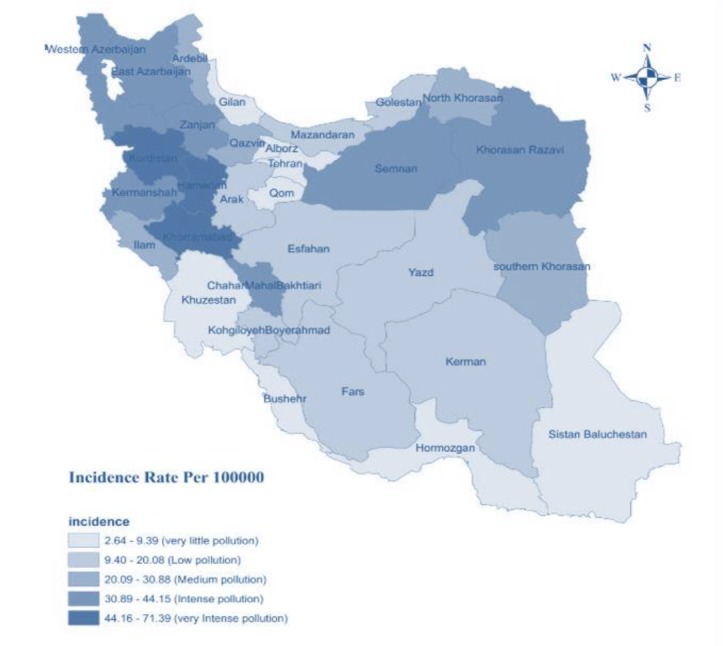
Average incidence rate of brucellosis in Iran during (2011-2018)

The first three provinces with the highest incidence rate of brucellosis, were as follows: Kurdistan (71.39 per 100,000), Lorestan (68.09 per 100,000) and Hamadan (56.24 per 100,000). Moreover, the first three provinces with the lowest incidence rate of brucellosis were as follows; Tehran (2.94 per 100,000), Gilan (2.64 per 100,000) and Hormozgan (5.53 per 100, 000). The distribution of seven-year incidence rate of brucellosis in the provinces of Iran graphically reported in Figure 1.

Table 1 shows the summary descriptive characteristics of annual incidence of brucellosis cases, in 2014 the highest incidence rate was observed, also during 2011 the lowest incidence rate was reported.

To compare the performance of the SVM, MARS and RF time series models, the RMSE, MAE and ICC statistics in the testing sets were calculated (Table 2). The minimum RMSE and MAE values were observed in the MARSE model (0.09 and 0.065 respectively), also the largest R^2^ (0.924) was related to MARSE model. Among four models, the RF model was outperformed the SVM and MARS models for the used data sets.

**Table 2:** Evaluation of the prediction Models over the test set

**Model**	**RMSE**	**MAE**	**MARE**	**R** ^2 ^
Random Forest	0.341	0.290	0.193	0.924
Support Vector Machine	0.384	0.238	0.176	0.717
Multivariate Adaptive Regression Splines	0.090	0.065	0.044	0.992
Auto Regressive Integrated Moving Average	0.566	0.489	0.288	0.370

**Figure 2:**
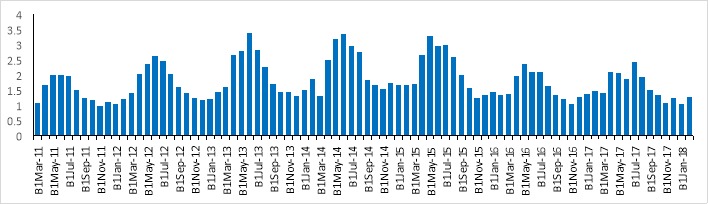
The time trend of incidence rate of brucellosis in Iran, during (2011-2018)

According to Figure 2, in May and Jul of each year, the peak of the incidence rate of brucellosis occurred. Moreover, an increasing trend was observed in the spring months, then decreasing cycle occurred, so that in winter months, the lowest incidence rate of brucellosis was observed.

**Figure 3:**
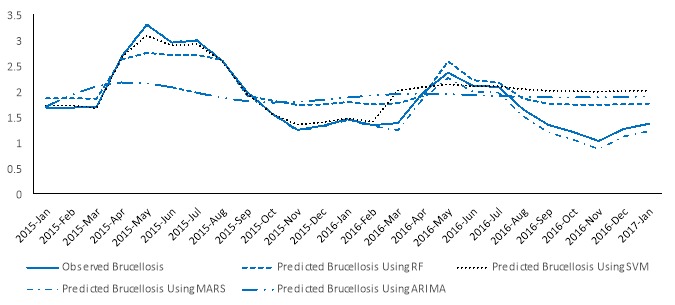
Prediction values for the test sets obtained using models along with the observed values

The temporal variation of observed incidence of brucellosis cases and their estimated values obtained from RF, SVM, MARS and ARIMA time series models for the test sets is plotted in Figure 3. The estimated value of brucellosis frequencies obtained from MARSE time series model was better than those of the other three models, especially for the peaks. Residual plots were also portrayed for the four methods (Figure 3). The performance of the MARSE model was better compared with the SVM, MARS, and ARIMA for and MARSE returned smaller residuals.

**Figure 4:**
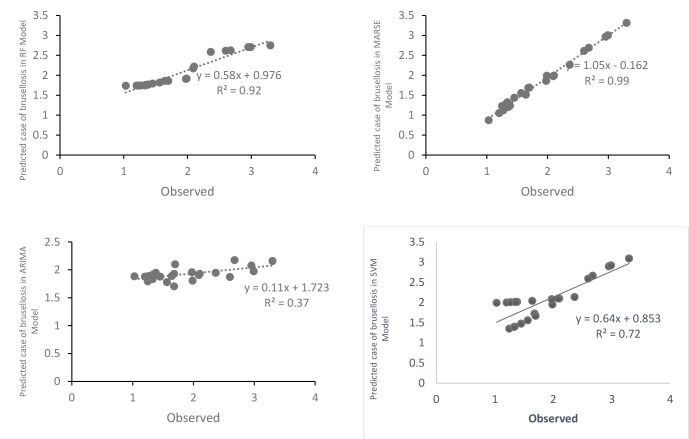
Comparison of Predictions vs. observed values using four models

The highest incidence rate of brucellosis was observed in May and Jun months (spring season) and the lowest incidence rate was observed in Nov and Jan months (the end of autumn and early periods of winter). Moreover, Figure 4 shows the scatter plot of the observed and estimated values of the monthly incidence of human brucellosis cases obtained from all four modes. The Y-axis and X-axis show the predicted and observed values of brucellosis frequency respectively. All points for MARSE model fall in the first quadrant where the direction of the estimated values coincided with the observed values, so the best prediction was seen in MARSE model (R^2^=0.99) and worst prediction was seen in the ARIMA model (R^2^=0.37).

## Discussion

In this national study (2011-2018), overall 118867 cases of brucellosis were observed. Moreover, incidence rate of brucellosis was 21.78 (per 100000). Due to some reasons, the incidence rate of brucellosis may be low reported and so underestimate. Firstly, all of our cases were those who referred to the health care centers, therefore some patients who came to the private clinic were not included in this study. Secondly, the signs and symptoms of brucellosis (e.g. fever, shivering, sweating, weakness, fatigue, headache, lumbar pain and etc.) are very similar to influenza and may be misdiagnosed, or it may that patients do not refer in order to diagnosis^17^. In this study, the male-female ratio of patients was 1.43 and the majority of patients were 20-34 year old. Dastjerdi et al^18^ showed the male: female ratio was 2.1, and the brucellosis was most common in individuals aged 15-20 yr in Isfahan Province in Iran. This result is probably due to the fact that men in developing countries such as Iran are more involved in livestock-related activities (veterinarians, livestock farmers, shepherd, slaughterhouse workers, predators, etc.) than women. Moreover, young men had more contact with livestock and animal products, especially in rural areas. Mamani et al. showed similar results^19^.

Furthermore, the present study showed that the three western provinces of Iran (Kurdistan, Lorestan, and Hamadan) had located in high-pollution of brucellosis (i.e. Incidence rate of brucellosis ≥ 44 cases per 100,000). This finding is very logical since the mentioned provinces are agricultural poles, major livestock producers and dairy products. Our study showed the peak of the incidence rate of brucellosis occurred in May and Jul of each year.

In addition, an increasing trend and decreasing brucellosis trend were observed in spring months and winter respectively. The most important reason for this finding can be related to the time of livestock labor in terms of delivery and livestock in the spring and summer. Brucellosis occurred most frequently in grasslands at a moderate elevation where sheep and goats were the dominant livestock, and in years with cooler spring^20^.

Cold springs provide a suitable environment for the propagation of *Brucella* bacteria due to more rainfall and higher humidity. On the other hand, they prepare the environment for high birth rates and increased risk of disease.

According to our study, the change in incidence rate of brucellosis from 2011-2018, showed a decreasing trend, so incidence rate from 22.07 in 2014 reached 16.2 per 100,000 in 2018. Although the decreasing changes from three last years (2016-2018) were insignificant.

Because, brucellosis is still a major public health problem in Iran, especially in the western provinces. The most important challenges with controlling the disease in Iran can be included incorrect eating habits^21^, such consumption of non-pasteurized dairy products, as well as consumption of colostrum in the delivery season, especially in rural areas^22^. The consumption of unpasteurized dairy products increases the odds of brucellosis ^23, 24^. There was a positive history of consumption of cottage cheese (76%), fresh cow milk (30%) or other unpasteurized dairy products in brucellosis cases^25^. Finally, our study showed that MARSE model had the best forecast and ARIMA model (as the classical approach) had the worst prediction. The strengths of data-mining models are as follow as it can handle non-linear relationships between predictors and outcome and have the simplicity of interpretation as well as^9^.

The present study had some limitations. We could not evaluate the role of environmental effects on the incidence rate of disease due to the lack of access to the monthly incidence rate of brucellosis by provinces. Moreover, due to the same reasons, we were not able to study the role of important risk factors on the occurrence of brucellosis, such as; the history of contact with the infected animal, the history of exposure to contaminated dairy products, and etc.

## Conclusion

The present study indicated brucellosis was more common in males, 25-29 aged yr, western provinces and spring months. The disease had a decreasing trend in the last years. MARS model was more appropriate rather than data mining models for prediction of monthly incidence rate of brucellosis.

## Acknowledgements

We would like to thank the staffs of the Department of Zoonotic Diseases at the Center for Communicable Disease Control, Ministry of Health and Medical Education of Iran (MOHME). Moreover, we thank the Vice-Chancellor of Research and Technology of Hamadan University of Medical Sciences.

## Conflict of interest

None declared.

## Funding

This study (ID: IR.UMSHA.REC.1397.799) was funded by the Vice-Chancellor of Research and Technology of Hamadan University of Medical Sciences. The funders had no role in study design, data collection, and analysis, decision to publish, or preparation of the manuscript.

## Highlights

- Seven-year incidence rate of brucellosis in Iran was 21.78 (95% CI: 21.66, 21.91).
- Brucellosis was more common in males, western provinces and spring months.
- Brucellosis had a decreasing trend in the last years.
- Data-mining models are more appropriate approach for prediction of Brucellosis.

## References

[1] Franco MP, Mulder M, Gilman RH, Smits HL (2007). Human brucellosis. Lancet Infect Dis.

[2] Pappas G, Papadimitriou P, Akritidis N, Christou L, Tsianos EV (2006). The new global map of human brucellosis. Lancet Infect Dis.

[3] Chalabiani S, Nazari MK, Davoodi NR, Shabani M, Mardani M, Sarafnejad A, Amirzargar AA (2019). The Prevalence of Brucellosis in Different Provinces of Iran during 2013–2015. Iran J Public Health.

[4] Mostafavi E, Asmand M (2012). Trend of Brucellosis in Iran from 1991 to 2008. IJE.

[5] Esmaeili H (2015). Brucellosis in Islamic republic of Iran. J Med Bacterio.

[6] Singh B, Dhand NK, Gill J (2015). Economic losses occurring due to brucellosis in Indian livestock populations. Prev Vet Med.

[7] Poester FP, Gonçalves VtSP, Lage AP (2002). Brucellosis in Brazil. Vet Microbiol.

[8] Lee HS, Her M, Levine M, Moore GE (2013). Time series analysis of human and bovine brucellosis in South Korea from 2005 to 2010. Prev Vet Med.

[9] Han J, Pei J, Kamber M. Data mining: concepts and techniques: Elsevier; 2011.

[10] Sapankevych NI, Sankar R (2009). Time series prediction using support vector machines: a survey. IEEE Comput Intell Mag.

[11] Liaw A, Wiener M (2002). Classification and regression by randomForest. R news.

[12] Segal MR. Machine Learning Benchmarks and Random Forest Regression. San Francisco: UCF; 2004.

[13] Svetnik V, Liaw A, Tong C, Culberson JC, Sheridan RP, Feuston BP (2003). Random Forest: A Classification and Regression Tool for Compound Classification and QSAR Modeling. J Chem Inf Comput Sci.

[14] James G, Witten D, Hastie T, Tibshirani R. An introduction to statistical learning: Springer; 2013.

[15] Kisi O (2015). Pan evaporation modeling using least square support vector machine, multivariate adaptive regression splines and M5 model tree. J Hydrol.

[16] Sharda V, Patel R, Prasher SO, Ojasvi P, Prakash C (2006). Modeling runoff from middle Himalayan watersheds employing artificial intelligence techniques. Agric Water Manag.

[17] Hajia M, Rahbar M, Keramat F (2009). Epidemiological, clinical, diagnostic and treatment aspects of hospitalized Brucellosis patients in Hamadan. Ann Trop Med Public Health.

[18] Dastjerdi MZ, Nobari RF, Ramazanpour J (2012). Epidemiological features of human brucellosis in central Iran, 2006–2011. Public health.

[19] Mamani M, Majzoobi MM, Keramat F, Varmaghani N, Moghimbeigi A (2018). Seroprevalence of brucellosis in butchers, veterinarians and slaughterhouse workers in Hamadan, Western Iran. J Res Health Sci.

[20] Li Y-J, Li X-L, Liang S, Fang L-Q, Cao W-C (2013). Epidemiological features and risk factors associated with the spatial and temporal distribution of human brucellosis in China. BMC infectious diseases.

[21] Keramat F, Karami M, Alikhani MY, Bashirian S, Moghimbeigi A, Adabi M (2019). Cohort profile: Famenin brucellosis cohort study. J Res Health Sci.

[22] Eini P, Keramat F, Hasanzadehhoseinabadi M (2012). Epidemiologic, clinical and laboratory findings of patients with brucellosis in Hamadan, west of Iran. J Res Health Sci.

[23] Sofian M, Aghakhani A, Velayati AA, Banifazl M, Eslamifar A, Ramezani A (2008). Risk factors for human brucellosis in Iran: a case–control study. Int J Infect Dis.

[24] Nematollahi S, Ayubi E, Karami M, Khazaei S, Shojaeian M, Zamani R (2017). Epidemiological characteristics of human brucellosis in Hamadan Province during 2009–2015: results from the National Notifiable Diseases Surveillance System. Int J Infect Dis.

[25] Khani Y, Mollajan A, Rahimi F (2015). Inappropriate dietary and occupational patterns: major risk factors associated with brucellosis in the area covered by Karaj Health Center No 2. Int J Enteric Pathog.

